# Parallel deep transcriptome and proteome analysis of zebrafish larvae

**DOI:** 10.1186/1756-0500-6-428

**Published:** 2013-10-24

**Authors:** Magnus Palmblad, Christiaan V Henkel, Ron P Dirks, Annemarie H Meijer, André M Deelder, Herman P Spaink

**Affiliations:** 1 Center for Proteomics and Metabolomics, Leiden University Medical Center, Zone L04-Q, P.O. Box 9600, 2300 RC, Leiden, The Netherlands; 2 ZF-screens B.V., J.H. Oortweg 19, 2333 CH, Leiden, The Netherlands; 3 Institute of Biology, Leiden University, Einsteinweg 55, 2333 CC, Leiden, The Netherlands

## Abstract

**Background:**

Sensitivity and throughput of transcriptomic and proteomic technologies have advanced tremendously in recent years. With the use of deep sequencing of RNA samples (RNA-seq) and mass spectrometry technology for protein identification and quantitation, it is now feasible to compare gene and protein expression on a massive scale and for any organism for which genomic data is available. Although these technologies are currently applied to many research questions in various model systems ranging from cell cultures to the entire organism level, there are few comparative studies of these technologies in the same system, let alone on the same samples. Here we present a comparison between gene and protein expression in embryos of zebrafish, which is an upcoming model in disease studies.

**Results:**

We compared Agilent custom made expression microarrays with Illumina deep sequencing for RNA analysis, showing as expected a high degree of correlation of expression of a common set of 18,230 genes. Gene expression was also found to correlate with the abundance of 963 distinct proteins, with several categories of genes as exceptions. These exceptions include ribosomal proteins, histones and vitellogenins, for which biological and technical explanations are discussed.

**Conclusions:**

By comparing state of the art transcriptomic and proteomic technologies on samples derived from the same group of organisms we have for the first time benchmarked the differences in these technologies with regard to sensitivity and bias towards detection of particular gene categories in zebrafish. Our datasets submitted to public repositories are a good starting point for researchers interested in disease progression in zebrafish at a stage of development highly suited for high throughput screening technologies.

## Background

In recent years there have been tremendous advances in transcriptome and proteome technologies. For transcriptome analysis the use of deep sequencing of RNA samples (RNA-seq) has shown to be an excellent method to obtain unbiased datasets that correlate well with microarray analyses [1]. As to the depth of sequences that can be analyzed it is clear that transcriptome sequencing is currently hardly limited by sequencing hardware, whereas this is still not the case for proteomic studies; however, also in the latter case there are rapid advances in mass spectrometry methods for high-throughput analyses [2-4]. Although these technologies are currently applied to many research questions in various model systems ranging from cell cultures to entire organism level, there are few comparative studies of these technologies in the same system. Therefore, there is still little quantitative information on how various transcriptome and proteomic technologies compare as to their sensitivities towards rarely expressed genes and the dynamic range of detection of genes expressed at various levels. In this study we chose the zebrafish model [5] to compare state of the art transcriptome and proteome analyses. We have used samples derived from the same batches of larvae to compare deep sequencing of mRNA with a fast mass spectrometrical ion trap technology.

## Methods

### Zebrafish larvae

Zebrafish were handled in compliance with local animal welfare regulations and maintained according to standard protocols (http://zfin.org). The breeding of adult fish was approved by the local animal welfare committee (DEC) of the University of Leiden, The Netherlands. All protocols adhered to the international guidelines specified by the EU Animal Protection Directive 86/609/EEC. Ten parent zebrafish couples were kept separately from one another for mating the following week. Embryos were grown at 28°C in egg water (60 μg/ml Instant ocean sea salt, Sera Marin). The egg water was refreshed every day. At 5 DPI, approximately 200 embryos per condition were collected and snap-frozen in liquid nitrogen. The frozen embryos were ground to homogeneity with a pipet tip and split into two portions that were stored at −80°C. One part was used for RNA sample preparation and the other part for protein sample preparation.

### RNA isolation

Embryos were homogenized in 1 ml of TRIzol reagent (Invitrogen), and total RNA was extracted according to the manufacturer’s instructions. RNA samples were treated with DNaseI (Ambion) to remove residual genomic DNA. RNA integrity was analysed on an Electrophoresis BioAnalyzer (Agilent Technologies). The average RIN value of the RNA samples was 9.7 with a minimum of 9.5.

### Microarrays

For microarrays analysis 500 ng total RNA was combined with Spike A and amplified according to the Agilent Two-Color Microarray-Based Gene Expression Analysis guide version 5.5 (G4140-90050, Agilent technologies). In order to exclude dye bias we have labelled all used sample either with Cy3 or Cy5 label. Amino-allyl modified nucleotides were incorporated during the aRNA synthesis (2.5 mM rGAC (GE Healthcare), 0.75 mM rUTP (GE Healthcare), 0.75 mM AA-rUTP (TriLink Biotechnologies). Synthesized aRNA was purified with the E.Z.N.A. MicroElute RNA Clean Up Kit (Omega Bio-Tek). The quality was inspected on the BioAnalyzer (Agilent Technologies) with the Agilent RNA 6000 kit (5067–1511, Agilent Technologies). 5 μg of aRNA was dried down and dissolved in 50 mM carbonate buffer pH 8.5. Individual vials of Cy3/Cy5 from the mono-reactive dye packs (GE Healthcare) were dissolved in 200 μl DMSO. To each sample, 10 μl of the appropriate CyDye dissolved in DMSO was added and the mixture was incubated for 1 h. Reactions were quenched with the addition of 5 μl 4 M hydroxylamine (Sigma-Aldrich). The labeled aRNA was purified with the E.Z.N.A. MicroElute RNA Clean Up Kit. Yields of aRNA and CyDye incorporation were measured on the NanoDrop ND-1000 (Thermo Scientific).

Each hybridization mixture was made up from 825 ng Cy3 and 825 ng Cy5 material. Hybridization mixtures were made as described in the Agilent Two-Color Microarray-Based Gene Expression Analysis guide version 5.5 (G4140-90050, Agilent technologies). The samples were loaded onto 4x180k D. rerio micro-arrays (Design ID:028233, Agilent Technologies) and hybridized for 17 hours at 65°C. Afterwards the slides were washed and scanned (20 bit, 3 μm resolution) in an ozone-free room with the Agilent G2505C scanner as described in the Agilent Two-Color Microarray-Based Gene Expression Analysis guide version 5.5 (G4140-90050, Agilent technologies). Data was extracted with Feature Extraction (v10.7.3.1, Agilent Technologies) with the GE2_107_Sep09 protocol for two-color Agilent micro-arrays.

Micro-array data was processed using Rosetta Resolver 7.2 (Rosetta Biosoftware). The raw micro-array data have been deposited in the NCBI GEO database as part of a larger experiment directed at studying disease progression accepted as project GSE44226. Gene expression values were obtained from the control sample channels (two Cy3, two Cy5) using the limma package for R/Bioconductor [6]. Raw data were background-corrected, after which the channels containing infection sample were replaced by the control sample from the same array, resulting in two identical data sets per array. These data were loess and quantile normalized within and between arrays, respectively [7].

### Transcriptome sequencing

The same RNA as used for the microarray analysis was used for constructing libraries for RNA deep sequencing. A total of 3 μg of RNA was used to make RNA-Seq libraries using the Illumina TruSeq RNA Sample Preparation Kit v2 (Illumina Inc., San Diego, USA). In the manufacturer’s instructions two modifications were made. In the adapter ligation step 1 μl instead of 2.5 μl adaptor was used. In the library size selection step the library fragments were isolated with a double Ampure XP purification with a 0.7x beads-to-library ratio. The mRNA-seq library was sequenced single-end, with a read length of 51 nucleotides, in a single lane on an Illumina GAIIx sequencer according to the manufacturer's recommendations. Base-calling was performed by the Illumina pipeline. Low quality nucleotides were removed from the sequencing reads using the trim function of the CLC Genomics Workbench (version 4.0, CLC bio, Aarhus, Denmark). Subsequently, reads were aligned to the Zv9 zebrafish genome assembly annotated by Ensembl (http://www.ensembl.org, release 62) using the short read mapper implemented in the Genomics Workbench, allowing up to 1 mismatch per sequence. The deep sequencing data sets have also been deposited in the NCBI GEO database as a reference series GSE44352. The Perl and R scripts used in the data analysis are available on http://ms-utils.org/zebrafish.

### Proteomics

Zebrafish larvae were ground with a pestle in liquid nitrogen in 1.5 mL Eppendorf tubes and vortexed for 30 seconds in a lysis buffer consisting of 9 parts 20 mM Tris–HCl, pH 8.5, 20 mM NaCl and 1 part protease inhibitor cocktail (P 8340, Sigma-Aldrich). The samples were placed on a shaking table for 20 minutes at room temperature before spinning down cellular debris at 16,100 × *g* for 10 minutes at 4°C. The supernatant was transferred to a fresh tube and the supernatants as well as the remaining pellets frozen in −35°C. Five μL NuPAGE® LSD Sample Buffer (Invitrogen) was added to 20 μL, 20 μg protein, as measured by the bicinchoninic acid (BCA) protein assay (ThermoScientific), and loaded in each lane on a 10-lane 4-12% NuPAGE® Bis-Tris gel. The assembled Invitrogen XCell SureLock™ Mini-Cell was filled with SDS-MOPS Running Buffer and the separation run for 1 hour at 200 V. The gel was released from its casing and immersed in NuPAGE® SureStain Colloidal Blue (55 mL deionized water, 20 mL methanol, 20 mL “Stain A” and 5 mL “Stain B”) and left on a shaker overnight at room temperature. The staining solution was decanted and the gels were washed with deionized water before being scanned in an OptiGo UV imager (Isogen Life Science, De Meern, the Netherlands).

The gel lanes were cut into 48 identical slices fractions using a custom-made OneTouch Mount and Lane Picker (The Gel Company, San Francisco, CA). The gel slices were then removed with a Acu-Min® stainless steel Precision Probe with a 35° Single Bend Tip (#6) (Moody Tools Inc., Warwick, RI) and each slice placed into one well in a 96-well polypropylene PCR plate (Greiner Bio-One, Frickenhausen Germany). Using 50 and 250 μL Rainin 8-channel multi-pipettors, each gel slice was washed for 5 minutes at room temperature with 100 μL 25 mM ammonium bicarbonate (ABC), followed by a wash with 100 μL 30% acetonitrile in 25 mM ABC for 10 minutes and a final wash of 100 μL 80% acetonitrile in 25 mM ABC for 10 minutes, discarding the solution between each wash. Cystines were reduced by addition of 75 μL 10 mM DTT in 25 mM ABC and the plate incubated at 56°C for 20 minutes. After discarding the DTT solution, cysteines were alkylated by addition of 75 μL 55 mM iodoacetamide in 25 mM ABC with incubation at room temperature in darkness for 20 minutes. The gel pieces were washed again as above and the supernatant discarded. The proteins were digested in-gel by addition of 15 μL 5 ng/μL porcine trypsin (Promega, Madison, WI) and incubation for 6 hours at 37°C, after which the digestion was quenched with the addition of 1 μL 5% trifluoroacetic acid (TFA). The solution was removed, followed by a second extraction with 20 μL 0.1% TFA for 1 h and the solution pooled with the first extraction. The plate was stored at −35°C until analysis by LC-MS/MS.

All SDS-PAGE fractions were analyzed by LC-MS/MS using a splitless NanoLC-Ultra 2D plus (Eksigent, Dublin, CA) for parallel ultra-high pressure liquid chromatography (UHPLC) with an additional loading pump for fast sam-ple loading and desalting. The UHPLC system was configured with 300 μm-i.d. 5-mm PepMap C18 trap columns (Thermo Scientific, Sunnyvale, CA), 15-cm 300 μm-i.d. ChromXP C18 columns supplied by Eksigent and running 90-minute linear gradients from 4 to 33% acetonitrile in 0.05% formic acid. The UHPLC system was coupled on-line to an HCTultra PTM Discovery System ion trap (Bruker Daltonics, Bremen, Germany). After each MS scan, up to ten abundant multiply charged species in m/z 300–1300 were automatically selected for MS/MS but excluded for one minute after being selected twice. The UHPLC system was controlled using the HyStar3.4 with a plug-in from Eksigent and the HCTultra ion trap by esquireControl 6.2, all from Bruker.

Peptides and proteins were identified using Mascot 2.4 running on an 8-CPU in-house server and the *Danio rerio* UniProt database (20130814) and validated using PeptideProphet and ProteinProphet in the Trans-Proteomic Pipeline [8]. In the Mascot search, a peptide mass measurement tolerance of [−0.5, 2.5] Da was used with MS/MS mass measurement tolerance [−0.5, 0.5] Da and allowing one missed cleavage. Carbamidylation of cysteine was included as the only fixed modication and oxidation of methionine as the only variable modification. Protein names from UniProt were matched against the microarray transcript (gene) names, expanding each protein group to individual proteins to match as many genes as possible, bearing in mind that not all of these proteins or protein isoforms are independently quantified.

## Results and discussion

### Data collection

In order to compare transcriptome and proteome data from the same biological sample we used extraction procedures for RNA and proteins on a large batch of zebrafish embryos that were frozen 5 days after fertilization. We have chosen this time point since in our recently published high throughput disease screening assays for infectious disease we have used this time point to measure for bacterial proliferation and host responses [9,10]. In addition this time point is also used for the measurement of cancer progression in a zebrafish xenotransplant model [11]. For RNA analysis state of the art Agilent custom made 180,000 probes microarrays and Illumina deep sequencing technologies were compared. Raw datasets have been submitted to the NCBI GEO database (accession number GSE44226). For protein analysis, proteins were separated on SDS-PAGE. From the SDS-PAGE and LC-MS/MS, 1,694 proteins could be identified with a ProteinProphet minimum probability of 0.95 and a global false-discovery rate of 1%. Over half (1,224) of these proteins were supported by more than one unique peptide identification. The number of spectra matching peptides from a particular protein was used as a simple metric of the abundance of that protein, a procedure known as spectral counting [12]. In the accepted protein identifications, this number ranged from 1 to 7,444, with 627 proteins identified by 10 or more spectra. The raw data and Mascot results files are available in PRIDE [13] XML (accession number PXD000145).

### Data analysis

**Figure 1:**
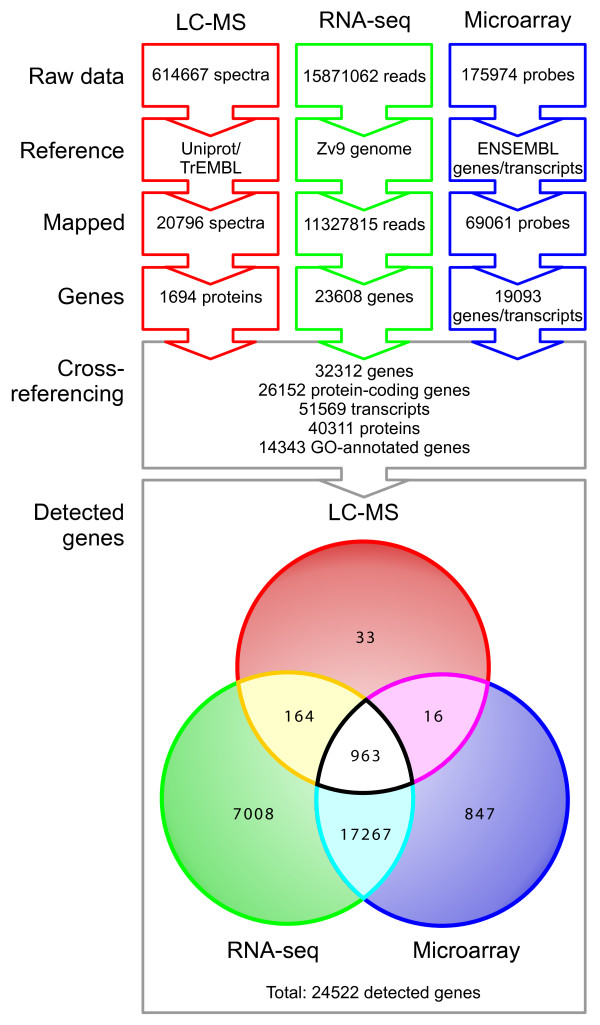
**Analysis flowchart.** Schematic overview of the analysis pipeline, and numbers of genes/proteins detected by each technology (top). With each technology, raw data are mapped to a suitable reference. In case of LC-MS/MS, the raw data is compared to known Uniprot/TrEMBL proteins. With RNA-seq, the reads are aligned to the Zv9 genome. Microarray probes were annotated with a known Ensembl gene or transcript. To make comparisons across technologies possible, the protein and array annotations were translated to Ensembl genes. The Venn diagram (bottom) shows the overlaps amongst the genes detected by the different technologies. Green: Illumina RNA-seq, Blue, Agilent microarray and Red, proteomic data from LC-MS/MS.

In Figure 1 we show a schematic overview of the analysis pipeline, and numbers of genes/proteins detected by each technology. With each technology, raw data are mapped to a suitable reference. In case of LC-MS/MS, the raw data correspond to 20,796 confidently identified spectra (FDR = 1%) mapped to 1,694 zebrafish proteins. With RNA-seq, 11.3 million out of 15.9 million reads aligned to 25,255 distinct loci on the Zv9 genome. However, for 1,647 genes, the alignments did not overlap with annotated exons (many of these are non-protein coding genes), leaving 23,608 genes with quantified expression. Finally, using a microarray, 69,061 probes of 175,974 were annotated with a known Ensembl gene or transcript. As multiple probes can assay expression for the same gene locus, the number of annotated genes for which an expression measure was obtained is reduced to 19,212. 119 of these are non-protein coding, leaving 19,093 genes for comparison with other technology.

Only in the quantification of RNA-seq, direct use is made of Ensembl information. To make comparisons across technologies possible, the protein and array annotations need to be translated to Ensembl genes. This was done using current Ensembl annotations downloaded through Biomart.

The Venn diagram in the bottom part of Figure 1 shows the overlaps amongst the genes detected by the different technologies. In total, 24,522 genes were detected by at least one technology. 963 were detected by all three technologies. The protein identifiers associated with 91 LC-MS/MS spectra were not included in the current Ensembl annotations.

### Comparison of analysis technologies

**Figure 2:**
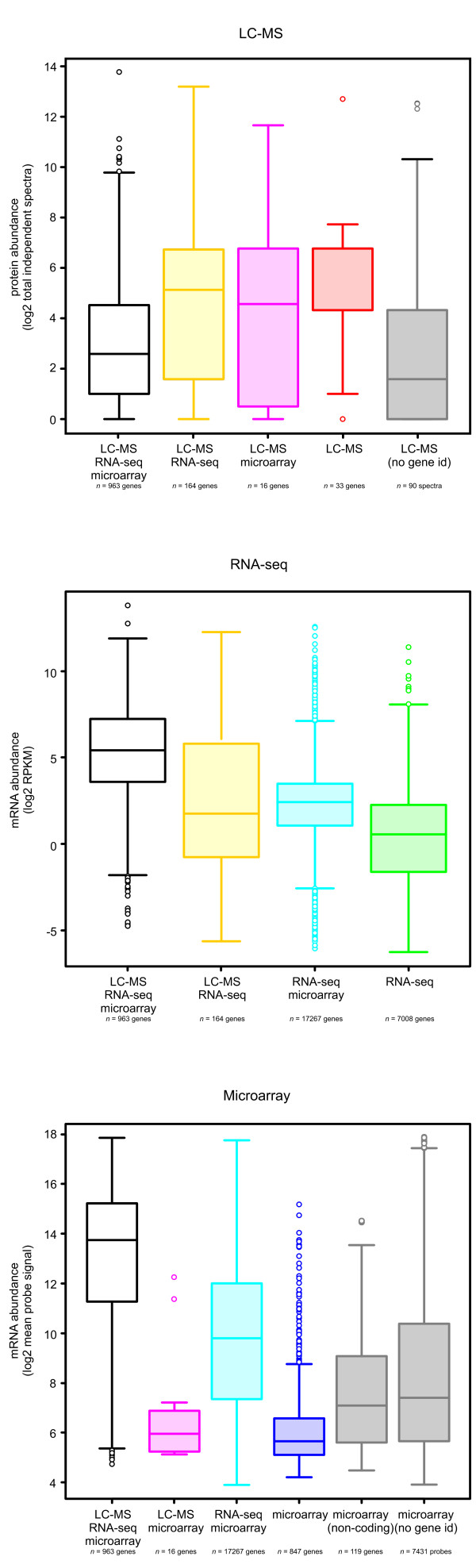
**Correspondence between detection technologies.** Boxplots of detection levels for each category in Figure 1 (the colours of the columns correspond to the categories in the Venn diagram). In general, genes detected using all technologies (first columns) have higher protein/mRNA abundance than genes detected using only a single technology (fourth columns). For LC-MS/MS and microarray, the last one and two columns, respectively, summarize signals that cannot be linked to Ensembl gene IDs.

**Figure 3:**
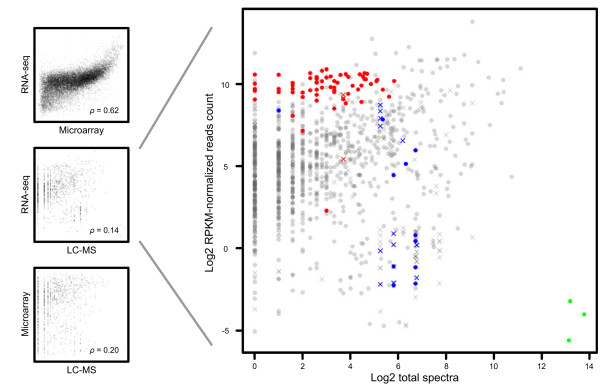
**Correlation between mRNA and protein detection signals.** Plots of transcript and protein detection levels for 963 genes detected by all three technologies (left). Every point represents a gene annotation. Correspondence is expressed by Spearman’s correlation coefficients (ρ). In the detailed view of the mRNA abundance assayed by RNA-seq versus protein abundance (right), three categories of proteins/transcripts that do not conform to the overall correlation trend are highlighted: ribosomal (red, GO cellular component ribosome), histones (blue, based on gene descriptions) and vitellogenins (green, based on gene descriptions). Crosses are multiple gene matches (one protein/many genes or many proteins/many genes).

Of each category shown in 7 colours in the Venn diagram of Figure 1 we have compared the detection levels in the boxplots in Figure 2. The total overlap of the annotated genes detected using all three technologies is shown in white. In general, these genes detected using all technologies (Figure 2, first columns) have higher protein/mRNA abundance than genes detected using only a single technology (Figure 2, columns 4-6). For LC-MS/MS and microarray, the last one and two columns, respectively, summarize signals that cannot be linked to Ensembl gene IDs. In the used custom microarray a majority of these probes were designed for exons that were either dubious or possibly linked to differential splicing. Future reannotation of the zebrafish genome will undoubtedly lead to removal of many of these probes from the design and therefore these were not analyzed further. It is of interest to note that the non-common overlap between the proteomics data is larger with the RNA-seq data (164 annotations in yellow) than with microarrays (16 annotations in pink), even though the expression levels are in the same range. This result emphasizes the advantage of an unbiased deep sequencing approach over a biased microarray approach in transcriptome analyses. We mostly focused on the overlapping set of 963 genes detected by all three technologies. As shown in Figure 3, microarrays and RNA-seq levels exhibit relatively high correspondence (Spearman’s correlation coefficient 0.62), but with a noticeable bias at high signal strengths. Correlation between the transcriptomic technologies and proteomics is less obvious, e.g. 0.14 for the RNA-seq *vs* MS. However, when we zoom in on the mRNA abundance assayed by mRNA-seq versus protein abundance in Figure 3B we can see that many genes that do not correlate in expression levels belong to three well known gene categories: ribosomal (red, GO cellular component ribosome), histones (blue, based on gene descriptions) and vitellogenins (green, based on gene descriptions). Based on the predicted functions of these groups of genes we can explain why there are such distinct differences at the transcription and proteome levels. Most obviously, the vitellogenins are maternally expressed proteins of which the genes are transcribed in the female liver and not in the embryos [14]. The vitellogenins are transported from the liver to the gonad and deposited in the eggs. Since the proteins are expected to be very stable in the embryo, a much higher level of the protein than the mRNAs is expected at 5 days post fertilization. The much higher level of ribosomal protein transcripts than protein levels can be explained because several of these also have a function as untranslated RNAs. Histones mRNAs are generally not polyadenylated, and therefore will be underrepresented in the RNA-seq data, because polyadenylated mRNA was captured using poly-dT primers prior to random-primed cDNA synthesis. In addition, histones are DNA-binding proteins with many positively charged amino acids, ionizing and fragmenting well in positive-mode electrospray-tandem mass spectrometry. Leaving out mappings to multiple genes increases the Spearman’s correlation between RNA-seq and MS data to 0.26, and by additionally leaving out the three identified and explained special cases it increases to 0.30.

## Conclusions

By comparing state of the art transcriptomic and proteomic technologies on samples derived from the same group of zebrafish embryos, we have for the first time benchmarked the differences in these technologies with regard to sensitivity and bias towards detection of particular gene categories in zebrafish. Our pipeline approach can be used by other groups to get a rapid insight into results obtained from different technology platforms. The results show that comparison of levels of RNA deep sequencing and proteomics have surprisingly high correlation if one considers the extreme differences between regulatory mechanisms at the level of transcription and translation. For such comparisons it does not seem necessary to include microarray analysis, which by its biased character was contributing little to the overall comparison. We can therefore conclude that the RNA sequencing and LC-MS/MS protein technologies used are extremely robust and can be used for more detailed analysis of the difference between the transcriptome and proteome levels. By focusing on the outliers in correlation plots, we can pinpoint translational and posttranslational regulatory mechanisms that underlie the observed differences for further study. The datasets that we have submitted to the public databases are a good starting point for other researchers that are interested in disease progression in zebrafish at a stage of development that is highly suited for read out using high throughput optical and genomics technologies [9,11,15]. We are currently using this approach to expand our datasets to a much deeper level at the proteomic level and to also include various zebrafish models for disease, including infectious disease and cancer.

## Competing interests

The authors declare that they have no competing interests.

## Authors' contributions

HPS and MP initiated the study. RPD prepared all samples. MP and AD performed the proteomics analyses and CVH compared the proteome and transcriptome data. HPS, AHM and AD provided materials and resources for this study. All authors contributed to writing the manuscripts, read and approved the final manuscript.
